# Exploring the need for sexually transmitted infection education among university student athletes in Saskatchewan

**DOI:** 10.14745/ccdr.v50i78a02

**Published:** 2024-07-24

**Authors:** Isabel Hedayat, Nicholas Steinberg, Sarah Akhtar, Adam T Clay, Danielle R Frost

**Affiliations:** 1 Department of Academic Family Medicine, University of Saskatchewan, Regina, SK

**Keywords:** sexually transmitted infections, knowledge, athletes, survey, Saskatchewan, universities, educational needs assessment, college

## Abstract

**Background:**

Sexually transmitted infections (STIs) are common in young adults in Canada and their prevalence is rising. Assessing sexual health knowledge among young adults is essential for developing effective STI education strategies. However, there is limited research on the sexual health knowledge of Canadian university athletes, who have increased risks of STIs.

**Objective:**

To determine perceived and objective levels of knowledge on STIs among university athletes and their preferred methods of knowledge translation regarding sexual health information.

**Methods:**

U SPORTS athletes at the University of Saskatchewan and the University of Regina were invited to complete an electronic survey between January–March 2022. Participants completed the Sexual Transmitted Disease Knowledge Questionnaire (STD-KQ) and self-reported their confidence in their answers. Participants were asked about testing beliefs, where they receive their sexual health information and their preferred format for STI information delivery.

**Results:**

One hundred participants completed the survey (14% response rate). Participants had a median composite self-reported STI knowledge score of 2.8 out of 5 (interquartile range [IQR]: 2.4–3.6). The median participant scored 12 out of 27 (44%) on the STD-KQ (IQR: 8–17). Sixty-seven percent of participants received sexual health information from a physician. Sixty-one percent of participants believed embarrassment may prevent them from getting tested or screened. The three most popular methods of health information sharing were online modules (34%), in-person lectures/conferences (24%) and self-paced videos (20%).

**Conclusion:**

This study highlighted that STI knowledge is limited in university athletes. Comprehensive online educational interventions may be effective at improving knowledge.

## Introduction

Sexually transmitted infections (STIs) are common in young adults in Canada and their prevalence is rising ((1)). With few exceptions, STIs are not vaccine-preventable. Though many STIs are asymptomatic and almost all are either treatable or curable, many complications can result from contracting an STI. These can include infertility, life-threatening complications and increased risk of cancer. Additionally, treatment-resistant STIs have emerged, which highlights the need for both education on STI treatment and prevention ((2)).

Research on the university and college student population has focused on sexual health behaviours and negative health outcomes ((3)). However, sexual health knowledge may impact sexual behaviour and health-seeking behaviour of young adults. Lack of knowledge may lead to delayed treatment ((4)), which can lead to complications. Few studies have focused on the sexual health knowledge among university students, but the literature suggests students outside of Canada generally have a low level of sexual health knowledge (5–7).

Relative to non-athletes, university and college athletes may be at an increased risk, as they report more sexual partners, unsafe sex and drinking before or during sex (8,9). A study from South Africa found significant limitations in knowledge about STIs in college athletes ((10)). English athletes in the SPORTSMART trial were found to engage in riskier sexual practices than age-matched counterparts who were not athletes ((9)). Limitations in knowledge and high-risk sexual activity may put university athletes at increased risk of contracting and transmitting STIs, compared to the general population.

Comprehensive sexual education can influence sexual practices ((11,12)). Assessing sexual health knowledge among young adults is essential for developing effective STI education strategies ((13)). To our understanding, there has been no published research on the sexual health knowledge of Canadian university athletes. As such, we sought to determine perceived and objective levels of knowledge on STIs among athletes at two Canadian universities. To inform the design of effective educational interventions, we also sought to determine sources of health information for athletes and their preferred methods of knowledge translation regarding sexual health information.

## Methods

All athletes governed by the national body for university sports (U SPORTS) at the University of Regina (n=314) and University of Saskatchewan (n=424) were invited to participate in an anonymous online survey in January 2022. Athletes, who were all registered students meeting course load requirements, were invited by e-mails sent by the Athletic Director (University of Saskatchewan) or by the student athletics website (University of Regina) to participate in the survey hosted on SurveyMonkey. Participants were informed of the option to participate in a prize draw for one of four $75 gift cards as an incentive for participation. A reminder to complete the survey was sent in February 2022. The survey was open until March 2022. Athletes participated in the survey remotely at the time and place of their choice, without investigators present.

The survey consisted of demographic questions, the objective Sexually Transmitted Disease Knowledge Questionnaire (STD-KQ, which is a collection of validated true/false questions to assess knowledge of STIs) ((14)), and questions to evaluate self-reported STI knowledge (which was presented using a five-point Likert scale [range of “1=not at all confident” to “5=very confident”]). Additionally, participants were asked about what they believe is involved in STI testing, what they perceived as barriers to getting tested for STIs and preferred methods of health education delivery.

Descriptive statistics were calculated. Intergroup comparisons were performed using a Mann-Whitney U test (knowledge score), Fisher exact test, or chi-squared test, as appropriate. Spearman rank correlation coefficients were calculated to determine the relationship between self-reported knowledge and STD-KQ scores. Multiple linear regression was performed to predict STD-KQ scores from demographic variables (gender of sport team, age and university). Scores from the STD-KQ were the total number of correct answers, with a score of zero for wrong and a score of one for correct. “Don’t know” answers were counted as wrong. All analysis was performed using the IBM SPSS version 28 statistical software. Since all athletes were invited to participate, a sample size was not calculated *a priori.*

This project was reviewed and approved by the University of Saskatchewan Research Ethics Board (Beh #3033) and received approval from athletics authorities at the University of Regina and the University of Saskatchewan prior to distribution. Participants provided consent by voluntarily starting the survey after being presented with a written informed consent form outlining its objective, how long it would take to complete and how personal information would be protected.

## Results

The survey was started by 105 participants (14% response rate). One hundred participants provided information on demographics, practices and beliefs. Ninety-four completed the STD-KQ questionnaire (13%). The median age of the participants was 20 years (interquartile range [IQR]: 19–22 years). Fifty-five (55%) participants played on women’s teams and 45 (45%) played on men’s teams. Thirty-four (34%) were students at the University of Regina and 65 (65%) were students at the University of Saskatchewan. Athletes participated in track and field/cross country (n=34; 34%), ice hockey (n=20; 20%), wrestling (n=11; 11%), volleyball (n=10; 10%), football (n=8; 8%), soccer (n=8; 8%), basketball (n=6; 6%) and swimming (n=3; 3%).

### Sexually transmitted infection knowledge

The median participant score on the STD-KQ was 12 out of 27 (44%; IQR: 8–17). A multiple regression was run to predict STD-KQ scores from gender of sports team, age and university. The variables did not predict knowledge (F(3,1.235), *p*=0.302, R^2^=0.040). Responses to individual STD-KQ items are provided in **Appendix**, **Table A1**. When the self-reported confidence was averaged across the different STI knowledge areas (transmission, prevention, etc.) the median composite score was 2.8 out of 5 (IQR: 2.4–3.6), which roughly corresponds to the ‘somewhat confident’ option. There was a weak correlation (r_s_=0.321, *p*=0.003) between average self-reported confidence in STI knowledge and objective STD-KQ scores.

### Sexually transmitted infection beliefs and sources of health information

Athletes reported that they would seek STI screening or testing in a variety of settings (**Table 1**). They also specified what tests they believed would be involved and outlined several potential barriers to getting tested (Table 1). Similarly, athletes sought general and sexual health information from multiple sources and preferred online modules to learn about health topics (**Table 2**).

**Table 1:** Sexually transmitted infection screening practices and testing beliefs

Question stem	Question response options	Women (n=55)	Men (n=45)	Total (n=100)	*p*-value (men vs. women)
Where would you go for STI screening and/or testing?	Family doctor's office	18 (33%)	8 (18%)	26 (26%)	0.071
	Sexual health clinic	15 (27%)	6 (13%)	21 (21%)	
	Walk-in clinic	13 (24%)	17 (38%)	30 (30%)	
	Not sure	7 (13%)	13 (29%)	20 (20%)	
	Buy online STI testing kits	1 (2%)	0 (0%)	1 (1%)	
	Other	1 (2%)	1 (2%)	2 (2%)	
What do you believe STI testing involves?	Urine tests	50 (91%)	38 (84%)	88 (88%)	0.322
	Blood tests	48 (87%)	36 (80%)	84 (84%)	0.324
	Detailed sexual history	36 (65%)	14 (31%)	50 (50%)	<0.001
	Pap smear	26 (47%)	7 (16%)	33 (33%)	<0.001
	Examination of reproductive organs	22 (40%)	17 (38%)	39 (39%)	0.821
	Urethral swab	19 (35%)	12 (27%)	31 (31%)	0.397
	Semen tests	16 (29%)	10 (22%)	26 (26%)	0.436
	Rectal swab	15 (27%)	7 (16%)	22 (22%)	0.159
What might prevent you from getting STI screening and/or testing?	Embarrassment/uncomfortable conversations	36 (65%)	25 (56%)	61 (61%)	0.313
	Don’t know where to go	18 (33%)	23 (51%)	41 (41%)	0.063
	Difficulty in getting an appointment	18 (33%)	8 (18%)	26 (26%)	0.090
	Worries that someone (partner(s), friends, family) would find out	16 (29%)	15 (33%)	31 (31%)	0.648
	Lack of time during business hours	15 (27%)	9 (20%)	24 (24%)	0.397
	Breach of confidentiality (specifically within your sports/extracurricular activities)	12 (22%)	7 (16%)	19 (19%)	0.427
	Fear of invasive examination or testing (other than needles)	11 (20%)	6 (13%)	17 (17%)	0.377
	Fear of needles	4 (7%)	2 (4%)	6 (6%)	0.688
	Other	0 (0%)	2 (4%)	2 (2%)	0.198
	None of the above	6 (11%)	6 (13%)	12 (12%)	0.711

Abbreviation: STI, sexually transmitted infection

**Table 2:** Sources of health information

Question stem	Question response options	Women (n=55)	Men (n=45)	Total (n=100)	*p*-value (men vs. women)
Where do you get your health information from?	Your physician	44 (80%)	36 (80%)	80 (80%)	1.000
	Friends and family	36 (65%)	32 (71%)	68 (68%)	0.546
	Internet	33 (60%)	32 (71%)	65 (65%)	0.246
	Your physiotherapist	21 (38%)	27 (60%)	48 (48%)	0.030
	Other healthcare professional	20 (36%)	15 (33%)	35 (35%)	0.752
	Athletic therapist	18 (33%)	17 (38%)	35 (35%)	0.598
	Social media	12 (22%)	10 (22%)	22 (22%)	0.961
	TV, radio, podcasts	7 (13%)	7 (16%)	14 (14%)	0.685
	Coach	6 (11%)	5 (11%)	11 (11%)	0.974
	School	4 (7%)	2 (4%)	6 (6%)	0.688
	Advertisement such as billboards or posters	1 (2%)	0 (0%)	1 (1%)	1.000
	Scientific articles	0 (0%)	1 (2%)	1 (1%)	0.450
Where do you get your sexual health information from?	Your physician	36 (65%)	31 (69%)	67 (67%)	0.716
	Friends and family	29 (53%)	19 (42%)	48 (48%)	0.296
	Social media	17 (31%)	12 (27%)	29 (29%)	0.641
	Other healthcare professional	9 (16%)	11 (24%)	20 (20%)	0.150
	School	7 (13%)	5 (11%)	12 (12%)	0.804
	Internet	6 (11%)	9 (20%)	15 (15%)	0.268
	TV, radio, podcasts	6 (11%)	7 (16%)	13 (13%)	0.492
	Your physiotherapist	2 (4%)	0 (0%)	2 (2%)	0.500
	Athletic therapist	1 (2%)	2 (4%)	3 (3%)	0.587
	Nobody	1 (2%)	0 (0%)	1 (1%)	1.000
	Scientific articles	0 (0%)	1 (2%)	1 (1%)	0.450
	Coach	0 (0%)	0 (0%)	0 (0%)	-
What method of information delivery do you prefer for health education?	Online modules	14 (25%)	20 (44%)	34 (34%)	0.130
	Self-paced videos	13 (24%)	7 (16%)	20 (20%)	
	In-person lecture/conference	12 (22%)	12 (27%)	24 (24%)	
	In-person course	8 (15%)	4 (9%)	12 (12%)	
	Handouts	8 (15%)	2 (4%)	10 (10%)	

Abbreviation: -, not applicable

The results of this survey were used to propose recommendations for interventions aimed at improving STI knowledge and for linking university athletes with appropriate medical care (**Table 3**).

**Table 3:** Summary of potential interventions

Interventions	Considerations
Comprehensive STI education	· Online and self-paced video preferred o Some online courses exist but effectiveness has not been fully evaluated · Same education could be targeted to multiple athlete demographic groups · Provide athletes with the information, motivation and behavioural skills to enhance their sexual health (not just information aimed at avoiding negative health outcomes)
Digital literacy skills	· Digital literacy skills are needed to enable athletes to find accurate and unbiased sexual health information online
Access to clinical services	· Physicians were most frequent source of sexual health information · Preparticipation physicals may be an opportune time to provide STI screening and testing information · Provide athletes with a list of local sexual health services in that specific community/campus

Abbreviation: STI, sexually transmitted infection

## Discussion

Participants demonstrated low knowledge of STIs, as assessed by the STD-KQ. This study found that 20% of participants were unsure of where to go to get STI testing locally and many had false beliefs related to what this testing would involve. For example, 47% of female participants thought that STI testing included a Pap smear. Participants were asked to indicate, “I don’t know”, in their STD-KQ responses if they did not know the answer, but many incorrect responses were provided. This suggests that participants may be receiving and believing incorrect sexual health information, which highlights the need for reliable sources.

Previous studies have used a wide range of measurement tools and sexual health knowledge outcomes ((15)). This makes direct comparison challenging, but this study’s findings are consistent with previous studies that found gaps in knowledge about STI transmission and prevention ((10)). To the authors’ understanding, this is the first study identifying limited STI knowledge among Canadian university athletes. The results of this survey were used to propose recommendations for interventions aimed at improving STI knowledge and for linking university athletes with appropriate medical care (Table 3), which are described in more detail below.

Sports-based educational interventions have been found to increase STI knowledge and condom use ((16)). Our study provides information that can be used in designing effective educational interventions targeting athletes at Canadian universities. While many of the previous studies focused on a single-sex in a specific sport, this study found that the demographic variable did not predict STD-KQ scores. There was only a weak correlation between the objectively measured STD-KQ and self-reported STI knowledge. Considering this, an educational intervention could be targeted at university athletes, regardless of sex or self-perceived knowledge.

This is the first study to investigate what type of educational materials athletes prefer for STI information. The administered survey suggests that online modules and self-paced videos are popular in this population. This study’s results are consistent with others ((17)) in showing that young people use the internet to find general and sexual health information. This suggests that online modules might be effective tools for student athletes. Online sexual health education has been developed for some target groups, but the effectiveness of these interventions has not been fully evaluated ((18)). The high use of the internet for information-seeking also highlights the importance of teaching digital literacy skills to athletes to enable them to find accurate and unbiased sexual health information online.

Sexual health education programs are most effective when combined with access to clinical services ((19)). There were multiple potential barriers identified concerning STI screening and testing, many of which may have simple solutions. In terms of athletes not knowing where to go for testing, this could be addressed in a comprehensive educational intervention, or advertisements could be created that identify where these services are available within a community. Another option would be bringing testing services to athletes and students. For example, when testing kits and information were made readily available in a team’s change rooms, there was an increased identification of STIs and an increased ability to provide one-on-one counselling and treatment ((9,20,21)). However, this may be less effective in the setting used for this study, as participants identified embarrassment/uncomfortable conversations and fear of people finding out that they are getting STI testing as potential barriers. This study also showed that physicians were the top source of general and sexual health information for participants. Thus, a potential location for STI intervention could be during the preparticipation physical examinations completed by an athlete’s physician before competition, which would allow for more private access to sexual health resources. Inclusion of STI screening has been suggested (22), but is not included in guidelines for preparticipation physical examinations ((23)). One reason may be limited time during these preparticipation physicals, which already include many aspects of athlete health. However, it would take little time for a physician conducting a physical with an athlete to encourage the athlete to be screened for STIs regularly and to potentially provide the athlete with information or resources to then follow-up on in the near future.

## Limitations

There were some limitations to our research. While our survey was sent to all eligible athletes from two universities, there is the potential for non-response bias. The survey had respondents from each of the U SPORTS sports teams. However, the proportion of survey respondents from a particular sport did not match the proportion of U SPORT athletes overall (e.g., football players represented only 8.8% of survey respondents but represent 24% of U SPORT athletes at the two universities). U SPORT athletes are less ethnically diverse and women are underrepresented when compared to the overall population of Canadian universities ((24,25)). Thus, the results for this study should not be generalized to the overall student population. This study focused on levels of knowledge about STIs. Sexual health education should provide athletes with the information, motivation and behavioural skills to enhance their sexual health and not just information aimed at avoiding negative health outcomes ((19)). Additionally, it is assumed that self-reported preferences among students for types of educational materials will be reflected in their tendency to use these materials, as well as in their ability to retain adequate knowledge from them.

Future studies could be completed within other institutions in Canada to see if the findings are replicable. Future research could look at the uptake and effectiveness of various methods of providing sexual health education. Finally, another area of research could investigate whether these educational interventions result in changes in behaviour towards obtaining STI screening, testing and treatment for STIs and improving sexual wellbeing.

## Conclusion

Participants self-reported an intermediate knowledge and had a median score of 44% on the STD-KQ. A comprehensive online educational intervention may be effective at improving knowledge and sexual wellbeing, as would incorporate information about STI screening and testing into preparticipation physicals.

## Appendix

Survey questions are available upon request to the author at: drf575@usask.ca

**Table A1:** Responses to the sexual transmitted disease knowledge questionnaire

Question stem	Question response options	Women	Men	Total
Genital herpes is caused by the same virus as HIV	True	8 (15%)	2 (5%)	10 (11%)
	False^a^	16 (31%)	19 (45%)	35 (37%)
	Don't know	28 (54%)	21 (50%)	49 (52%)
Frequent urinary infections can cause chlamydia	True	3 (6%)	5 (12%)	8 (9%)
	False^a^	22 (42%)	9 (21%)	31 (33%)
	Don't know	27 (52%)	28 (67%)	55 (59%)
There is a cure for gonorrhea	True^a^	24 (46%)	21 (51%)	45 (48%)
	False	7 (13%)	1 (2%)	8 (9%)
	Don't know	21 (40%)	19 (46%)	40 (43%)
It is easier to get HIV if a person has another sexually transmitted disease	True^a^	14 (27%)	8 (19%)	22 (23%)
	False	13 (25%)	10 (24%)	23 (24%)
	Don't know	25 (48%)	24 (57%)	49 (52%)
Human papillomavirus (HPV) is caused by the same virus that causes HIV	True	8 (15%)	3 (7%)	11 (12%)
	False^a^	17 (33%)	14 (33%)	31 (33%)
	Don't know	27 (52%)	25 (60%)	52 (55%)
Having anal sex increases a person’s risk of getting hepatitis B	True^a^	15 (29%)	7 (17%)	22 (23%)
	False	8 (15%)	7 (17%)	15 (16%)
	Don't know	29 (56%)	28 (67%)	57 (61%)
Soon after infection with HIV a person develops open sores on his or her genitals (penis or vagina)	True	11 (21%)	1 (2%)	12 (13%)
	False^a^	18 (35%)	16 (38%)	34 (36%)
	Don't know	23 (44%)	25 (60%)	48 (51%)
There is a cure for chlamydia	True^a^	35 (67%)	29 (69%)	64 (68%)
	False	6 (12%)	1 (2%)	7 (7%)
	Don't know	11 (21%)	12 (29%)	23 (24%)
A woman who has genital herpes can pass the infection to her baby during childbirth	True^a^	29 (58%)	18 (44%)	47 (52%)
	False	4 (8%)	1 (2%)	5 (5%)
	Don't know	17 (34%)	22 (54%)	39 (43%)
A woman can look at her body and tell if she has gonorrhea	True	3 (6%)	2 (5%)	5 (5%)
	False^a^	27 (52%)	16 (38%)	43 (46%)
	Don't know	22 (42%)	24 (57%)	46 (49%)
The same virus causes all of the sexually transmitted diseases	True	0 (0%)	0 (0%)	0 (0%)
	False^a^	44 (85%)	30 (71%)	74 (79%)
	Don't know	8 (15%)	12 (29%)	20 (21%)
Human papillomavirus (HPV) can cause genital warts	True^a^	11 (21%)	11 (27%)	22 (24%)
	False	4 (8%)	0 (0%)	4 (4%)
	Don't know	37 (71%)	30 (73%)	67 (72%)
Using a natural skin (lambskin) condom can protect a person from getting HIV	True	15 (29%)	12 (29%)	27 (29%)
	False^a^	13 (25%)	10 (24%)	23 (24%)
	Don't know	24 (46%)	20 (48%)	44 (47%)
Human papillomavirus (HPV) can lead to cancer in women	True^a^	21 (40%)	16 (38%)	37 (39%)
	False	4 (8%)	0 (0%)	4 (4%)
	Don't know	27 (52%)	26 (62%)	53 (56%)
A man must have vaginal sex to get genital warts	True	2 (4%)	2 (5%)	4 (4%)
	False^a^	40 (77%)	31 (74%)	71 (76%)
	Don't know	10 (19%)	9 (21%)	19 (20%)
Sexually transmitted diseases can lead to health problems that are usually more serious for men than women	True	4 (8%)	3 (7%)	7 (7%)
	False^a^	25 (48%)	16 (38%)	41 (44%)
	Don't know	23 (44%)	23 (55%)	46 (49%)
A woman can tell that she has chlamydia if she has a bad smelling odour from her vagina	True	19 (37%)	7 (17%)	26 (28%)
	False^a^	12 (23%)	14 (33%)	26 (28%)
	Don't know	21 (40%)	21 (50%)	42 (45%)
If a person tests positive for HIV the test can tell how sick the person will become	True	2 (4%)	1 (2%)	3 (3%)
	False^a^	39 (75%)	26 (62%)	65 (69%)
	Don't know	11 (21%)	15 (36%)	26 (28%)
There is a vaccine available to prevent a person from getting gonorrhea	True	6 (12%)	0 (0%)	6 (6%)
	False^a^	22 (42%)	14 (33%)	36 (38%)
	Don't know	24 (46%)	28 (67%)	52 (55%)
A woman can tell by the way her body feels if she has a sexually transmitted disease	True	8 (15%)	4 (10%)	12 (13%)
	False^a^	29 (56%)	16 (39%)	45 (48%)
	Don't know	15 (29%)	21 (51%)	36 (39%)
A person who has genital herpes must have open sores to give the infection to his or her sexual partner	True	10 (19%)	9 (21%)	19 (20%)
	False^a^	27 (52%)	12 (29%)	39 (41%)
	Don't know	15 (29%)	21 (50%)	36 (38%)
There is a vaccine that prevents a person from getting chlamydia	True	2 (4%)	1 (2%)	3 (3%)
	False^a^	26 (50%)	18 (43%)	44 (47%)
	Don't know	24 (46%)	23 (55%)	47 (50%)
A man can tell by the way his body feels if he has hepatitis B	True	4 (8%)	1 (2%)	5 (5%)
	False^a^	16 (31%)	17 (40%)	33 (35%)
	Don't know	32 (62%)	24 (57%)	56 (60%)
If a person had gonorrhea in the past, he or she is immune (protected) from getting it again	True	0 (0%)	1 (2%)	1 (1%)
	False^a^	31 (60%)	17 (40%)	48 (51%)
	Don't know	21 (40%)	24 (57%)	45 (48%)
Human papillomavirus (HPV) can cause HIV	True	2 (4%)	2 (5%)	4 (4%)
	False^a^	12 (23%)	14 (33%)	26 (28%)
	Don't know	38 (73%)	26 (62%)	64 (68%)
A man can protect himself from getting genital warts by washing his genitals after sex	True	4 (8%)	3 (7%)	7 (7%)
	False^a^	26 (50%)	17 (40%)	43 (46%)
	Don't know	22 (42%)	22 (52%)	44 (47%)
There is a vaccine that can protect a person from getting hepatitis B	True^a^	35 (67%)	19 (45%)	54 (57%)
	False	7 (13%)	4 (10%)	11 (12%)
	Don't know	10 (19%)	19 (45%)	29 (31%)

^a^ Correct response
